# NF‐κB‐dependent secretome of senescent cells can trigger neuroendocrine transdifferentiation of breast cancer cells

**DOI:** 10.1111/acel.13632

**Published:** 2022-06-02

**Authors:** Clotilde Raynard, Xingjie Ma, Anda Huna, Nolwenn Tessier, Amélie Massemin, Kexin Zhu, Jean‐Michel Flaman, Florentin Moulin, Delphine Goehrig, Jean‐Jacques Medard, David Vindrieux, Isabelle Treilleux, Hector Hernandez‐Vargas, Sylvie Ducreux, Nadine Martin, David Bernard

**Affiliations:** ^1^ Centre de Recherche en Cancérologie de Lyon, Inserm U1052, CNRS UMR 5286, Centre Léon Bérard Université de Lyon Lyon France; ^2^ Department of Intensive Care The Affiliated Hospital of Yangzhou University, Yangzhou University Yangzhou China; ^3^ University of Lyon, CarMeN Laboratory INSERM, INRA, INSA Lyon, Université Claude Bernard Lyon 1 Bron France

**Keywords:** aging, breast cancer, cellular senescence, neuroendocrine transdifferentiation, senescence‐associated secretory phenotype

## Abstract

Cellular senescence is characterized by a stable proliferation arrest in response to stresses and the acquisition of a senescence‐associated secretory phenotype, called SASP, composed of numerous factors including pro‐inflammatory molecules, proteases, and growth factors. The SASP affects the environment of senescent cells, especially during aging, by inducing and modulating various phenotypes such as paracrine senescence, immune cell activity, and extracellular matrix deposition and organization, which critically impact various pathophysiological situations, including fibrosis and cancer. Here, we uncover a novel paracrine effect of the SASP: the neuroendocrine transdifferentiation (NED) of some epithelial cancer cells, evidenced both in the breast and prostate. Mechanistically, this effect is mediated by NF‐κB‐dependent SASP factors, and leads to an increase in intracellular Ca^2+^ levels. Consistently, buffering Ca^2+^ by overexpressing the CALB1 buffering protein partly reverts SASP‐induced NED, suggesting that the SASP promotes NED through a SASP‐induced Ca^2+^ signaling. Human breast cancer dataset analyses support that NED occurs mainly in p53 WT tumors and in older patients, in line with a role of senescent cells and its secretome, as they are increasing during aging. In conclusion, our work, uncovering SASP‐induced NED in some cancer cells, paves the way for future studies aiming at better understanding the functional link between senescent cell accumulation during aging, NED and clinical patient outcome.

## Graphical abstract

Senescent cells, which can accumulate during aging or after stresses, secrete many factors. The pro‐inflammatory NF‐kappaB‐dependent secretome induces neuroendocrine transdifferentiation of breast cancer epithelial cells.

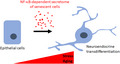

**Abbreviations**
CHG A/B: chromogranine A/B
CKI: cyclin‐dependent kinase inhibitor
ER: estrogen receptor
GO: gene ontology
GSEA: gene set enrichment analysis
METABRIC: molecular taxonomy of breast cancer international consortium
NBC: neuroendocrine breast carcinomas
NED: neuroendocrine differentiation
4‐OHT: 4‐HydroxyTamoxifen
SA‐β‐gal: senescence‐associated‐β‐galactosidase
SASP: senescence‐associated secretory phenotype
SCG: secretogranin
SR: super repressor

## 1 INTRODUCTION

Cellular senescence is a cell response triggered by various stresses, such as oncogenic or genotoxic stresses, as well as telomere shortening. Senescent cells thus accumulate during aging and/or following chronic exposure to factors inducing these stresses. This cell state corresponds to a stable cell cycle arrest and to the acquisition of some specific features, including an enhanced senescence‐associated‐β‐galactosidase (SA‐β‐gal) activity (Dimri et al., 1995) and the secretion of numerous factors, named senescence‐associated secretory phenotype or SASP (Acosta et al., 2008; Coppé et al., 2008; Kuilman et al., 2008). This SASP is mainly composed of metalloproteases, growth factors, and pro‐inflammatory molecules. Its composition notably depends on the signaling and transcription factors involved. So far, two main transcription factors are known to regulate levels of pro‐inflammatory SASP components: NF‐κB and C/EBPβ (Acosta et al., 2008; Chien et al., 2011; Kuilman et al., 2008), and NOTCH1 has been reported to induce a TGF‐β‐dependent secretome (Hoare et al., 2016). Senescence, largely through its SASP, plays key roles in regulating a variety of pathophysiological conditions including aging and cancer, aging increasing the risk of developing a cancer (Campisi & d'Adda di Fagagna, 2007; He & Sharpless, 2017; Smetana et al., 2016).

The SASP was reported to exert initial anti‐tumoral effects in autocrine and paracrine manners, by blocking cell proliferation and by recruiting and activating immune cells to eliminate harmful and defective senescent cells (Acosta et al., 2008; Kuilman et al., 2008; Kang et al., 2011). Nevertheless, the SASP can also have long term pro‐tumoral effects as it promotes, among others, tumor initiation, migration, epithelial‐mesenchymal transition (EMT), cancer stemness, and tumor resistance (Coppé et al., 2010; Kang et al., 2011; Krtolica et al., 2001; Liu & Hornsby, 2007). Hence, our understanding of the broad effects of the SASP in cancer and aging biology is emerging.

Neuroendocrine transdifferentiation (NED) has been depicted in some epithelial tumors, that allows to epithelial cells to possess neuronal and endocrine features. In particular, prostate epithelial cancer cells can undergo NED and prostatic cancers featuring neuroendocrine transdifferentiation are characterized by the presence of neuroendocrine cell foci into malignant cells. Neuroendocrine cells produce numerous pre‐hormones or peptide factors, such as chromogranine B (CHGB) and secretogranins (e.g., SCG2) (Lloyd, 2003). Androgen deprivation used in therapy against prostate cancer has been described as one of the main inducers of the emergence of a neuroendocrine phenotype, which is associated with a worse prognosis (Zhang et al., 2018). Though NED has also been depicted in other types of epithelial cancers, for instance, in breast cancer, very little is known about this process in this context (Inno et al., 2016; Lavigne et al., 2018; Makretsov et al., 2003). Breast neuroendocrine tumors are a rare and underrecognized subtype of mammary carcinomas, as they represent only 2%–4% of breast tumors. These tumors express specific NED markers, such as chromogranin A (CHGA) and SCG2 (Gallo et al., 2020). However, their mechanisms of formation and the features of these tumors in terms of treatment response, progression, and aggressiveness are largely debated and unknown (Gallo et al., 2020).

While assessing the effects of the SASP on breast cancer cells, we discovered a new link between senescent cells and their secretome, and the induction of NED in hormone‐dependent breast cancers during aging.

## 2 RESULTS

### 2.1 The SASP promotes neuroendocrine transdifferentiation (NED) of some breast cancer cells

To investigate the role of the SASP on some breast cancer cells, we produced control or SASP‐containing conditioned media. The SASP is released from normal human fibroblasts exposed to classical stresses, either through the activation of oncogenic proteins (Acosta et al., 2013; Collin et al., 2018) or using genotoxic drugs (Contrepois et al., 2017), provoking their entry into senescence. Here, MRC‐5 cells were infected with a retroviral vector to stably express a 4‐hydroxy tamoxifen (4‐OHT) inducible RAF protein, RAF:estrogen receptor (ER) (MRC‐5/RAF:ER). As expected, RAF activation by adding 4‐OHT into MRC‐5/RAF:ER led to cellular senescence. This was evidenced by (i) an increase in the senescence‐associated‐β‐galactosidase (SA‐β‐Gal) activity (Figure S1a), and (ii) in the mRNA level of the p21 cyclin‐dependent kinase inhibitor (CKI) (Figure S1b), as well as (iii) a decrease in the mRNA of the proliferation marker Ki67 (Figure S1c) and by (iv) an increase in the mRNA of the IL6, IL8, BMP‐2, and SPP1 SASP components (Figure S1d). This latter increases in SASP mRNA resulted in higher levels of SASP proteins in the supernatant, as illustrated for IL‐6 (Figure S1e).

**FIGURE 1:**
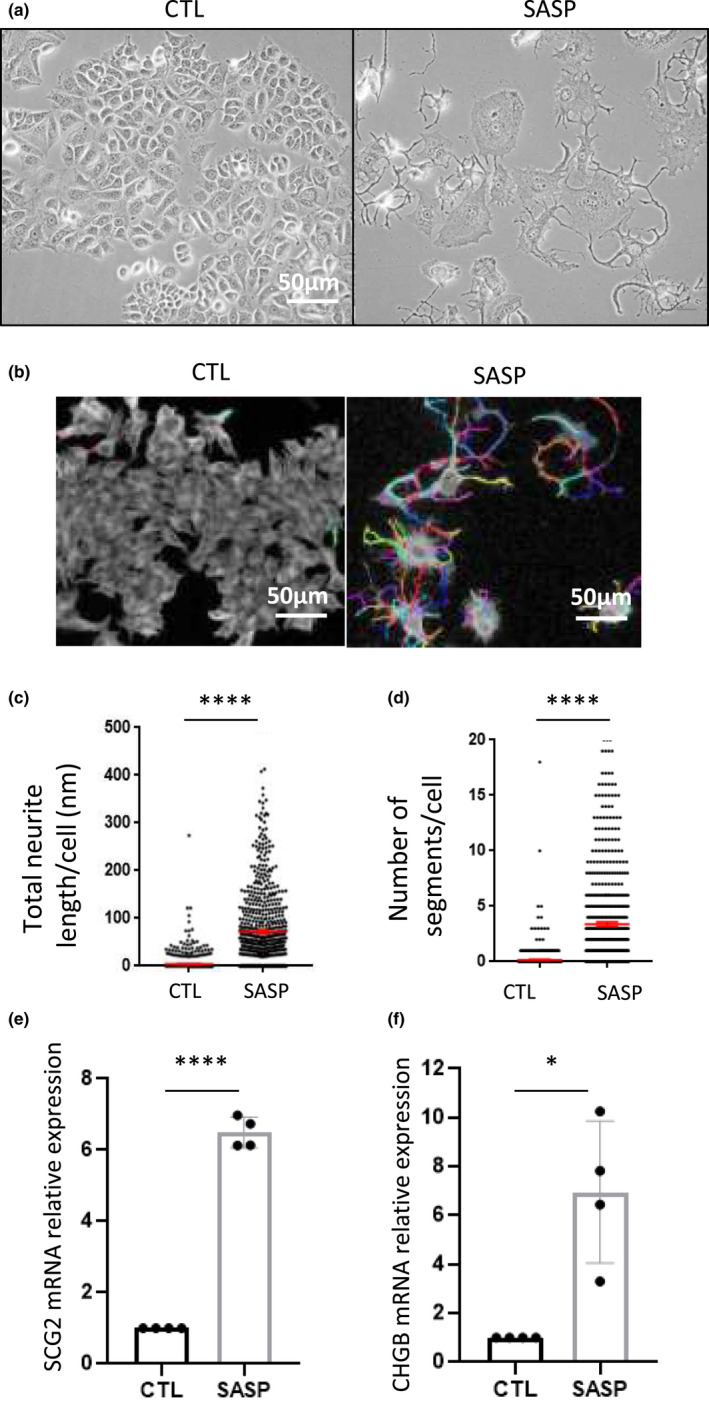
SASP promotes neuroendocrine transdifferentiation in MCF‐7 breast cancer cells. MCF‐7 cells were treated every 3 days with control (CTL) or SASP conditioned medium for 6 days. (a) Bright‐field micrographs of MCF‐7 cell morphology (representative of more than 5 independent experiments). (b) immunostaining against α‐tubulin and staining of nuclei with Hoechst were performed. High content imaging Operetta system was used to automatically acquire images and Columbus software was used to perform analysis of cell shape (colorful segments). (c) total neurite length per cell and (d) number of segments per cell were quantified (*n* = 3, unpaired non‐parametric Mann–Whitney *t*‐test, mean ± SEM). (e–f) qPCR was performed to quantify mRNA levels of the neuroendocrine markers (NEM) (e) SCG2 and (f) CHGB (*n* = 4, one sample *t*‐test, mean ± SEM)

Having validated the generation of SASP through this experimental set‐up, control (CTL, from MRC‐5/RAF:ER untreated with 4‐OHT) and SASP conditioned media were added to several breast cancer cell lines with molecular features representative of breast cancer cells: MCF‐7 (ER+; WT p53), T47D (ER+; mutated p53), or MDA‐MB231 (ER‐; mutated p53). For T47D (Figure S2a) and MDA‐MB‐231 (Figure S2b), we did not observe significant differences between control and SASP‐treated cells according to cell density and cell morphology. Strikingly in MCF‐7 cells, the SASP treatment resulted in decreased cell density, cellular senescence (Figure S2d–f), and strong morphological changes characterized by the appearance of ramifications arising from cell bodies (Figure 1a), reminiscent of neuroendocrine‐like structures. α‐tubulin staining highlighted the cytoskeleton containing the “neurite‐like” structures, which could be quantified using image analysis algorithms from the Columbus software (Figure 1b). This automated method revealed that total neurite length and number of segments per cell increased following treatment with SASP in MCF‐7 cells (Figure 1c,d). In addition to the neurite‐like structures, we assessed the expression of neuroendocrine markers, namely SCG2 and CHGB, and observed their upregulation in SASP‐treated MCF‐7 cells (Figure 1e,f). To further confirm and extend these unexpected results, we verified whether a SASP generated by another senescence inducer could trigger this neuroendocrine transdifferentiation (NED) in MCF‐7 cells, and whether this phenotype could also arise following exposure to SASP in prostate cancer cells known to be able to undergo NED. Here, we generated the SASP by treating WI‐38 fibroblasts with the genotoxic drug etoposide to activate pro‐inflammatory molecules, as previously described (Contrepois et al., 2017). We observed the induction of NED in MCF‐7 cells incubated in this SASP‐containing supernatant, as evidenced by changes in cell morphology and expression of NED markers (Figure S3a–d). In addition, we tested the SASP obtained from MRC‐5/RAF:ER on p53 functional LNCaP prostate cancer cells, well‐known to undergo NED upon certain stresses (Chang et al., 2014; Zhang et al., 2018; Zhu et al., 2014). Consistently, we observed that the SASP induced a NED in these cells according to both cell morphology and NED marker expression (Figure S3e–h), whereas the SASP did not induce this phenotype in p53 dysfunctional DU145 and PC‐3 prostate cancer cells (Figure S3i).

To know whether this NED is dependent on p53 activity, we knocked‐down p53 in MCF‐7 cells and we treated them with the SASP. p53 decrease did not prevent MCF‐7 entry in cellular senescence according to cell density and SA‐β‐Gal assays but partially impaired NED (Figure S2c–g).

Together, these results support that the secretome of senescent cells, the SASP, triggers a NED in some breast and prostate cancer cells, which correlates to p53 functionality.

### 2.2 NF‐κB‐dependent SASP factors drive induction of NED

The SASP is composed of a large variety of molecules, including but not limited to pro‐inflammatory molecules, growth factors, and proteases. Notably, the NF‐κB family of transcription factors is critical for promoting the expression of numerous pro‐inflammatory factors in senescent cells, including IL6, whereas they do not affect the expression of other types of SASP factors, such as BMP‐2 and SPP1 (Ferrand et al., 2015; Hoare et al., 2016). IL6 is a key pro‐inflammatory molecule from the SASP as it mediates some of the previously described paracrine SASP effects (Kuilman et al., 2008; Mosteiro et al., 2018). Moreover, it was reported to induce NED in LNCaP cells (Chang et al., 2014; Zhu et al., 2014).

**FIGURE 2:**
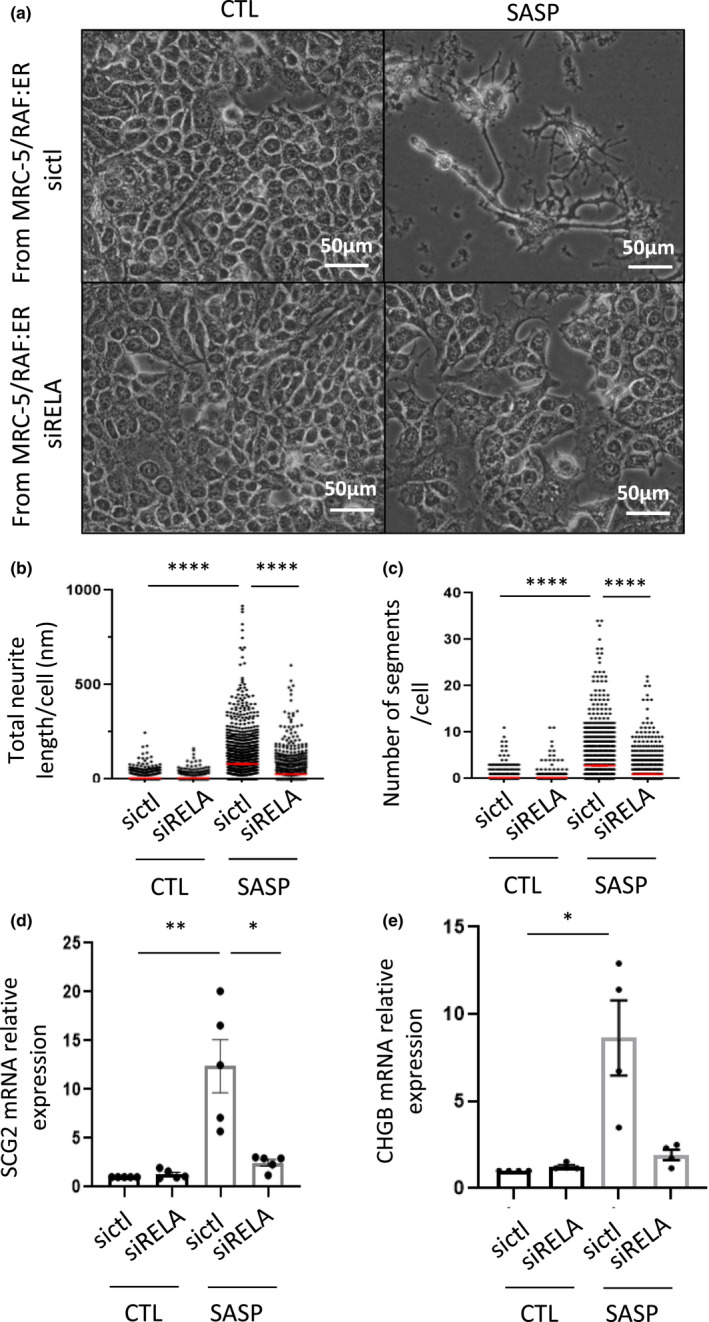
NF‐κB‐dependent pro‐inflammatory SASP molecules drive NED in MCF‐7. Six days after treatment with conditioned medium from MRC‐5/RAF:ER cells (CTL or SASP) transfected with control siRNA (sictl) or siRNA directed against RELA (siRELA), images of MCF‐7 cells (a) were taken. Neurite‐like structures were quantified, either (b) total neurite length or (c) number of segments per cell, using an Operetta system and Columbus software after immunostaining against α‐tubulin (*n* = 1408 cells, 3 independent experiments, non‐parametric Kruskal–Wallis, mean ± SEM). (d–e) RT‐qPCR on NEM (d) SCG2 and (e) CHGB were performed (n = 5 or 4, unpaired parametric Mann–Whitney *t*‐test, mean ± SEM)

We thus sought to determine whether removing the NF‐κB‐dependent pro‐inflammatory factors of the SASP would impact SASP‐induced NED. To this end, MRC‐5/RAF:ER cells were transfected with siRNA directed against RELA, a critical member of the NF‐κB family of transcription factors, or by stably expressing an inhibitor of the NF‐κB pathway, IκBα super‐repressor (SR), before activating RAF by adding 4‐OHT. We first confirmed knockdown of RELA in these cells (Figure S4a) and the subsequent decrease in the expression of NF‐κB‐dependent SASP factors including IL6 and IL8 (Figure S4b). Conversely, other markers of cellular senescence, such as SA‐β‐Gal activity and expression of p21 CKI, were not impacted, nor were NF‐κB‐independent SASP factors BMP‐2 and SPP1 (Figure S4c–f). Compared with the complete SASP, this NF‐κB‐independent SASP was less able to promote neurite‐like structures (Figure 2a–c) and to induce the expression of the NED markers, SCG2 and CHGB (Figure 2d–e). Similar results were obtained for the NED of MCF‐7 cells when the SASP applied was depleted of its NF‐κB‐dependent components by expressing an IκBα super‐repressor (SR) (Ferrand et al., 2015), an inhibitor that sequesters NF‐κB transcription factors in the cytoplasm, in MRC‐5/RAF:ER cells (Figure S5a–f).

Collectively, these data demonstrate that the NF‐κB dependent pro‐inflammatory SASP drives NED induced by the secretome of senescent cells.

### 2.3 Ca^2+^ as a secondary messenger drives SASP‐mediated NED

**FIGURE 3:**
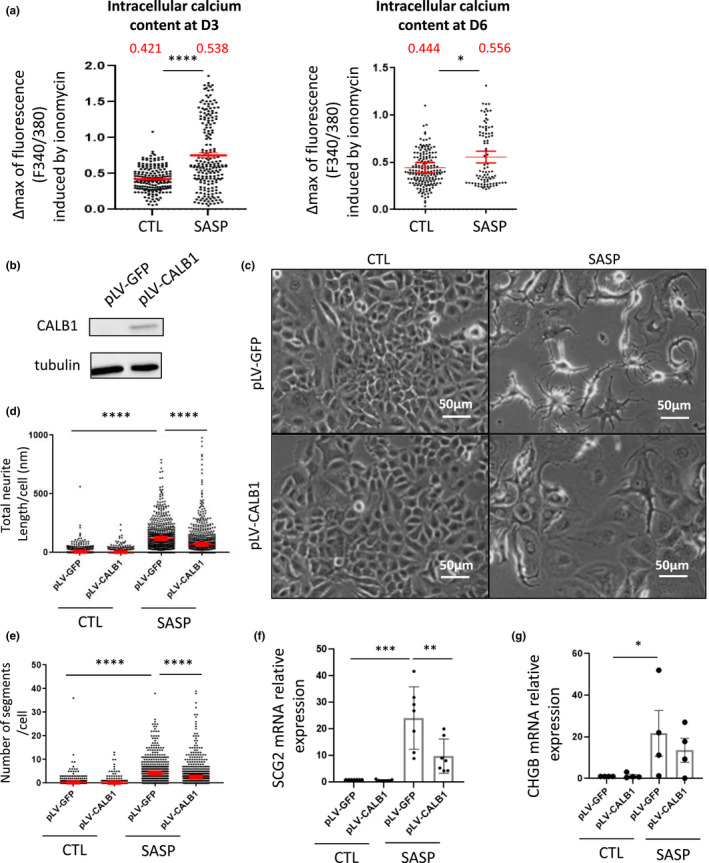
Ca^2+^ signaling mediates SASP‐induced NED. Three or 6 days after treatment with conditioned medium from MRC‐5/RAF:ER (CTL or SASP), (a) total intracellular Ca^2+^ content was assessed with the ratiometric probe Fura2‐AM after ionomycin stimulation (*n* = 194 for CTL and *n* = 205 for SASP at D3; *n* = 181 for CTL and *n* = 91 for SASP at D6, 3 independent experiments, non‐parametric Mann–Whitney *t*‐test, mean [in red] ± 95% of confidence). (b–g) MCF‐7 cells were infected with a lentiviral vector encoding CALB1 (pLV‐CALB1) or GFP (pLV‐GFP) as control vector. (b) CALB1 protein levels were validated by Western blot. After 6 days of treatment with MRC‐5/RAF:ER (CTL or SASP), (c) images of MCF‐7 were taken and (d,e) neurite‐like structures (total neurite length and number of segments per cell) quantified using the Operetta system and Columbus software after α‐tubulin immunostaining (*n* = 881 cells, 3 independent experiments, non‐parametric Kruskal–Wallis test, mean ± SEM). (f–g) RTqPCR on NEM (f) SCG2, and (g) CHGB were performed (n = 7 or 5, unpaired parametric Mann–Whitney *t*‐test, mean ± SEM)

Ca^2+^ is known to be a powerful secondary messenger, as it is activated by many ligand‐receptor couples and plays critical roles in numerous biological and cellular processes, such as cell shape, proliferation, migration, or cell death (Clapham, 2007; Humeau et al., 2018). Ca^2+^ fluxes have been associated with NED of prostate cancer cells (Mariot et al., 2002). Using the Fura2‐AM ratiometric calcium probe, we observed increased calcium in intracellular stocks (i.e., mitochondria and endoplasmic reticulum) (Figure 3a) with little variations in the cytosol (Figure S6a), in MCF‐7 after SASP treatment. Noteworthy, up‐regulation of Ca^2+^ observed in the stocks was abolished when the SASP was produced from NF‐κB‐deficient cells (Figure S6b) or when the SASP was applied to MCF‐7 depleted in p53 (Figure S6c). To investigate whether Ca^2+^ was involved in SASP‐induced NED, we used the CALB1 Ca^2+^ buffering protein (Kojetin et al., 2006; Noble et al., 2018) which binds intracellular calcium and balances its concentration (Kojetin et al., 2006; Schmidt, 2012). Its constitutive overexpression (Figure 3b) impaired NED induction by the SASP as evidenced by the reduction of neurite‐like structures (Figure 3c–e) and expression of the NED markers, SCG2, and CHGB (Figure 3f,g). In conclusion, these results support an involvement of calcium signaling in mediating, at least partly, SASP‐induced NED.

### 2.4 Human breast tumors displaying NED are ER+ and p53 WT and their proportion increases in older patients

**FIGURE 4:**
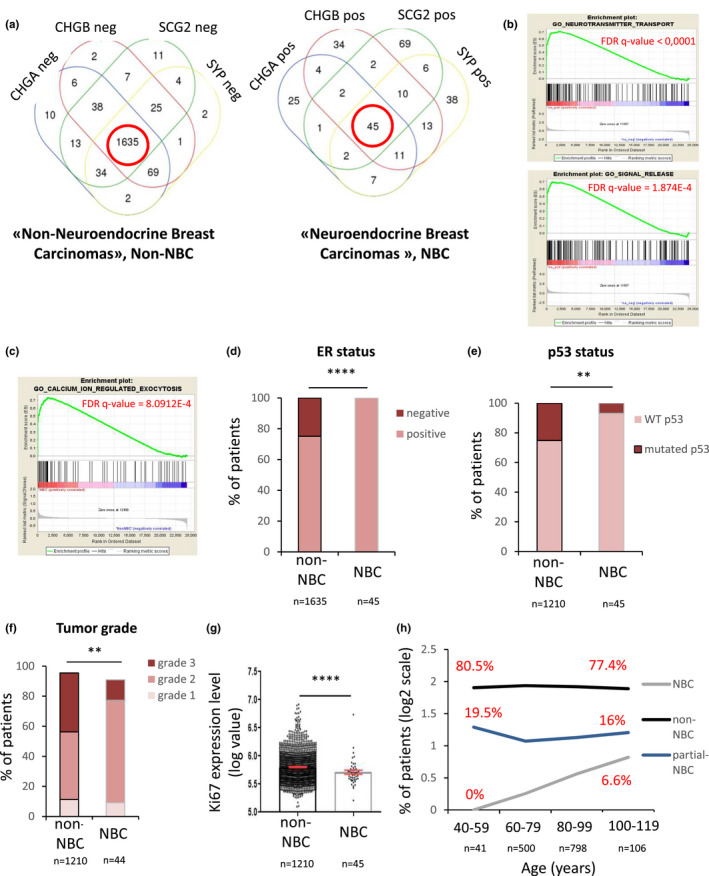
Breast tumors presenting neuroendocrine features are ER+, p53 wild‐type, and their proportion in breast cancer patients increases with aging. METABRIC data on breast tumor patients (clinical data and gene expression for 1904 tumors) were extracted and analyzed. (a) Based on the low (negative, neg) or high (positive, pos) expression of the four neuroendocrine markers (NEM) SCG2, CHGB, CHGA, and SYP, Venn diagrams were used to determine two groups of tumors: The “non‐Neuroendocrine Breast Carcinomas” (non‐NBC) (*n* = 1635) and the “Neuroendocrine Breast Carcinomas” (NBC) (*n* = 45). (b‐c) Gene set enrichment analysis (GSEA) between the two groups of patients. The y‐axis represents enrichment score (ES) and on the x‐axis are genes (vertical black lines) represented in gene sets from C5 MSigDB collection related to neuroendocrine features in (b) (“GO‐NEUROSTRANSMITTER TRANSPORT” and “GO_SIGNAL_RELEASE”) or in (c) CALCIUM signaling (CALCIUM_ION_REGULATED_EXOCYTOSIS”). FDR q‐Value is indicated in red. (d) ER status measured by immunohistochemistry was examined (*n* = 1635 for non‐NBC and *n* = 45 for NBC, Fisher's exact test). Further analyses were performed only on ER‐positive breast tumors. (e) Status of p53 (wild‐type, WT, or mutated) was analyzed in non‐NBC and NBC (*n* = 1210 for non‐NBC and *n* = 45 for NBC, Fisher's exact test). (f) Tumor grade was compared between non‐NBC and NBC patients (*n* = 1210 for non‐NBC and *n* = 45 for NBC, Fisher's exact test). (g) Mean mRNA levels of proliferation marker Ki67 in the two groups of patients was determined (*n* = 1210 for non‐NBC and *n* = 45 for NBC, non‐parametric unpaired Mann–Whitney *t*‐test). (h) Percentage of patients with the different types of tumors (NBC, non‐NBC or partial‐NBC presenting 1, 2, or 3 positive NEM) was examined in different age groups (*n* = 1445)

As mentioned above, the SASP did not induce marks of NED in breast cancer cells that were either ER‐negative or mutated for p53. To verify whether our findings are relevant for human breast tumors, we explored the METABRIC human breast cancer database (METABRIC Group et al., 2012). We isolated human breast tumors that displayed NED based on their co‐expression of four NED markers SCG2, CHGB, CHGA, and synaptophysin, SYP (Figure S7). Of the 1904 breast tumors identified in the database, 45 tumors were positive for all of these 4 markers (NBC for neuroendocrine breast carcinomas), within the expected range of 2–4%, whereas 1635 were negative for these 4 markers (non‐NBC) (Figure 4a). We then compared the gene expression profiles between NBC and the non‐NBC to define the specific features of these tumors. We performed Gene Set Enrichment Analysis (GSEA) based on the mean fold change in gene expression data between NBC and non‐NBC for all genes. As expected, molecular signatures such as neurotransmitter transport or signal release GO signatures were found in NBC compared with non‐NBC (Figure 4b). Moreover, some calcium GO signatures were upregulated exclusively in NBC patients, emphasizing the role of Ca^2+^ signaling in the neuroendocrine phenotype (Figure 4c). In line with the literature (Makretsov et al., 2003; Marchiò et al., 2017) and our data, NBC were all ER‐positive (Figure 4d). To avoid any bias during our analysis, we next compared features of NBC (all ER+) with ER+ non‐NBC, which represents luminal breast tumors and found that NBC were mostly p53 WT when compared with ER+ non‐NBC (Figure 4e). Moreover, NBC present lower proportions of grade 3 tumors (Figure 4f) and display a lower proliferation rate according to Ki67 expression levels (Figure 4g). These findings were reminiscent of the proliferation arrest observed during SASP‐induced NED *in cellulo*. Finally, the proportion of patients with a breast cancer displaying NBC strongly increased with age (Figure 4h). This is likely due to higher levels of SASP with aging, as senescent cells accumulate in older people (Fafián‐Labora & O'Loghlen, 2020; Karin et al., 2019; van Deursen, 2014).

## 3 DISCUSSION

The importance of the secretome of senescent cells in different pathophysiological contexts, including cancer biology, has been highlighted in the last 10 years (Sun et al., 2018). In this study, we uncover a new role for the secretome of senescent cells in shaping cancer cell identity, as their SASP induces NED of some epithelial cancer cells. Mechanistically, this effect of the SASP involves NF‐κB‐dependent SASP factors and relies on the induction of Ca^2+^ signaling. The ability of the SASP to induce NED in breast cancer cells seems to be relevant in vivo as it correlated with features of human neuroendocrine breast carcinoma (NBC) samples.

Several effects of the SASP are mediated by pro‐inflammatory molecules, in particular IL‐6 (Kuilman et al., 2008; Mosteiro et al., 2018). Interestingly, treatment with IL‐6 was described to induce NED in LNCaP prostate cancer cells (Yuan et al., 2007). We substantiated these findings, as the SASP, which is enriched in IL6, also induced NED in LNCaP cells (Figure S8a), arguing in favor of using IL6 to induce NED in breast cancer cells. Nevertheless, IL6 treatment of MCF‐7 cells did not induce NED (Figure S8b), suggesting either that factors inducing NED in breast and prostate cancer cells are different or that breast cancer cells are less permissive and need additional SASP factors to undergo NED. In line with the last hypothesis, eliminating the NF‐κB‐dependent pro‐inflammatory SASP, including IL6 and IL8, was sufficient to abolish SASP‐induced NED in breast cancer cells. This observation indicates that several NF‐κB‐dependent SASP factors are needed to drive NED of breast cancer cells.

Several pro‐inflammatory SASP factors are known to activate Ca^2+^ signaling by their binding and the activation of their receptors (Qiu et al., 1995; Rittner et al., 2006; Tuschil et al., 1992). Ca^2+^ signaling has also been reported in the regulation of NED in prostate cancer cells (Gackière et al., 2008; Mariot et al., 2002; Sekiguchi & Kawabata, 2019) and our analysis of the NBC in the METABRIC collection confirmed enrichment in Ca^2+^‐dependent signature. Our results support an involvement of the Ca^2+^ in mediating SASP‐induced NED of breast cancer cells as its level increased in cells undergoing NED and as its buffering repressed SASP‐induced NED. Of note, the Ca^2+^ increase is quite heterogenous in SASP‐treated cells, which may contribute to heterogeneity in the NED phenotype. In other words, not all the SASP‐treated cells show increased calcium and NED phenotype. In line with these observations, the molecular mechanisms increasing Ca^2+^ levels in response to the SASP, in particular, the channels involved, as well as the downstream mechanisms activated by calcium and leading to NED in response to the SASP, remain to be determined.

Little is known about NBC, leading to controversial conclusions about their origin and their behaviour, for instance in terms of prognosis, likely due to the scarcity of such breast tumors (Gallo et al., 2020; Marchiò et al., 2017). To better understand their features, we took advantage of the extensive number of breast cancer patients enrolled in the METABRIC database and of the genomic and clinical information available. As far as we know, this is the first time that such an approach is undertaken for NBC. The luminal WT p53 derived MCF‐7 breast cancer cell line displayed a NED phenotype after SASP treatment, whereas MCF‐7 cells depleted in p53, the luminal p53 mutant T47D or the triple negative p53 mutant MDA‐MB‐231 breast cancer cells displayed impaired or no NED phenotype. This seems to be particularly relevant as (i) our analysis of the METRABRIC database also supports that NBC are luminal p53 WT tumors and (ii) NBC have been reported to express the ER and to be eventually p53 WT and to be classified in the luminal breast cancer subtype according to the literature (Inno et al., 2016; Gallo et al., 2020; Marchiò et al., 2017). In line with the features of NED in SASP‐treated MCF‐7 cells, NBC compared with luminal non‐NBC tumors displayed a lower proliferation rate and consequently a lower tumor grade. Importantly, these analyses also show that patients displaying NBC are generally older. This is in line with our results showing that the secretome of senescent cells can induce NED as senescent cells and their secretome accumulate with aging (Karin et al., 2019; van Deursen, 2014).

One remaining question is whether NED and senescence can occur in the same cells. In line with previous results, the SASP can induce paracrine senescence in MCF‐7 cells where NED can also take place, suggesting that the two processes can occur in the same MCF‐7 cells. Still p53 depletion in MCF‐7 cells does not impact the SASP‐induced senescence whereas it partially rescues SASP‐induced NED suggesting that senescence by itself is not sufficient to trigger NED. Moreover, NBC do not display clear cut increase of senescence markers supporting that NED can occur in non‐senescent cells.

The events driving NBC formation, a rare event, instead of driving classical non‐NBC luminal breast cancer are largely unknown (Gallo et al., 2020). Overall, our work proposes a new hypothesis by which senescent cells and their secretome contribute to NED and NBC formation. More generally and beyond NBC, and as suggested by our results obtained in prostate cancer cells, our work paves the way for future studies investigating the contribution of senescent cells, especially during aging, to the formation of other types of neuroendocrine carcinoma and/or to the transdifferentiation of some adenocarcinoma into a neuroendocrine phenotype. This may ultimately lead to improving clinical applications in terms of NBC treatment and prognosis (Rubin et al., 2020).

## 4 MATERIAL AND METHODS

### 4.1 Vectors, transfection, and infection

The retroviral vector pBABE/RAF:ER expresses an inducible RAF protein. The lentiviral vectors pLV‐puro‐CALB1 expressing CALB1 and pLV‐GFP as control were used (VectorBuilder). The retroviral vector pBabe‐Puro‐IκBα super repressor (SR) (Plasmid #15291, Addgene) or its empty control was used. To transfect 293 T lentiviral‐producing cells and 293 GP retroviral‐producing cells, GeneJuice transfection reagent (Merck Millipore) was used according to the manufacturer's recommendations. Two days after transfection, the virus‐containing supernatant was mixed with fresh medium (1:20 for 293 T cells and 1:4 for 293 GP cells) and hexadimethrine bromide at 8 μg/mL (Sigma‐Aldrich) and used to infect target cells. One day later, infected cells were selected using puromycin (Invivogen) at 500 ng/ml for 4 days.

### 4.2 Cell culture, reagents, and conditioned medium production

MCF‐7, MDA‐MB231, T47D, LNCaP, PC‐3, DU145, WI‐38, and MRC‐5 cells (ATCC, Manassas, VA, USA), 293 T lentiviral‐ and 293 GP retroviral‐producing cells (Clontech, Mountain View, CA, USA) were cultured in Dulbecco's modified Eagle's medium (DMEM, Life Technologies) containing GlutaMax and supplemented with 10% fetal bovine serum (FBS, Sigma‐Aldrich) and 1% penicillin/streptomycin (ThermoFisher Scientific). Cells were maintained at 37°C under a 5% CO_2_ atmosphere. MRC‐5/RAF:ER were treated with 4‐OHT 100 nM, and the medium was changed after 3 days. Conditioned medium was collected 3 days later and filtered using 0.45 μm sterile filters (Clearline, 146561). This conditioned medium was diluted 1:5 or 1:10 with fresh medium to treat breast cancer or prostate cancer cells, respectively, every 3 days for 6 days.

SASP production in WI‐38 cell treated with etoposide was previously described (Contrepois et al., 2017). Shortly WI‐38 hTERT cells were grown in MEM (Invitrogen), 10% FBS, 1 mM sodium pyruvate, 2 mM L‐glutamine, and 0.1 mM non‐essential amino acids and penicillin–streptomycin (Sigma). Senescence was induced by treating cells with this medium containing 20 μM etoposide (Sigma) for 14 days with fresh medium every 3–4 days. After 14 days, cells were washed and incubated in fresh medium without etoposide for an additional 7 days. The medium in contact with cells during the last 3 days of culture was collected, centrifuged at 5000 × *g* for 5 min, and the supernatant was frozen at −80°C before its use.

For treatment with recombinant IL6 protein (Peprotech, 200–06), MCF‐7 and LNCaP cells were treated every 2 days with 20 ng/ml recombinant IL6 for 7 days.

### 4.3 siRNA transfection

For siRNA knockdown, we used an ONTARGETplus nontargeting siRNA control SMARTpool (sictl) or an ON‐TARGETplus siRNA SMARTpool targeting RELA (siRELA) or TP53 (sip53) (Horizon Discovery) (see Table S1 for their sequence). Cells were reverse transfected with siRNAs using Dharmafect 1 transfection reagent according to the manufacturer's instructions (Horizon Discovery). siRNAs were used at a final concentration of 15 nM. Sequences of the 4 siRNAs included in each pool are provided in Table S1. The day after transfection, medium was changed with DMEM with glutamax (10% FBS and 1% antibiotics).

### 4.4 Reverse transcription and real‐time quantitative PCR

NucleoZOL (Macherey‐Nagel) was used to perform total RNA extraction according to the manufacturer's recommendations. The First‐Strand cDNA Synthesis Kit (GE Healthcare, Chalfont St Giles, UK) was used to synthesize cDNA from 2 μg of total RNA, according to the manufacturer's instructions. The RT reaction mixture was diluted 1:4 and used as a cDNA template for qPCR. TaqMan quantitative PCR was carried out on a FX96 Thermocycler (Bio‐Rad). The PCR mixture contained TaqMan mix (Roche), 200 nM of primers (see Table S2 for their sequence), Universal Probe Library probe (100 μM, ThermoFisher Scientific) for the gene of interest (TaqMan Gene Expression Assays [Primers/probe], Life technologies), and 50 ng/μg of cDNA template. Reactions were performed in triplicate with the following program: 95°C 10 min, followed with 40 cycles of 95°C for 10 s, 59°C for 30 s. The relative amount of mRNA was calculated using the comparative Ct (ΔΔCT) method, following data normalization against GAPDH housekeeping gene.

### 4.5 Crystal violet staining and SA‐β‐galactosidase staining

For crystal violet, MCF‐7, T47D, DU145, PC‐3 and MDA‐MB231 were washed with PBS 1X, fixed for 15 min in 3.7% formaldehyde, washed with PBS 1X, and stained with crystal violet solution at 0.05% overnight. Scans of wells are presented.

For SA‐β‐galactosidase staining, MRC‐5/RAF:ER cells were washed once with PBS 1X, fixed for 5 min in 2% formaldehyde / 0.2% glutaraldehyde, rinsed twice with PBS 1X, and incubated overnight at 37°C in SA‐β‐Galactosidase staining solution as previously described (Debacq‐Chainiaux et al., 2009).

### 4.6 Immunofluorescent staining and quantification of neurite‐like structures

After 6 days of treatment with conditioned medium in 96‐well plates (PerkinElmer, 600,558), cells were fixed in paraformaldehyde 3.7% for 10 min and permeabilized with 0.1% triton X‐100 for 10 min, then blocked in PBS‐Tween 0.05% containing 20% FBS (PBST‐FBS). The antibody against α‐tubulin (1:1000; Sigma‐Aldrich T6199) was incubated overnight at 4°C in PBST‐FBS. After three washes using PBST‐FBS, cells were incubated for 1 h with anti‐rabbit IgG coupled with Alexa Fluor 455 (LifeTechnologies, A21424) diluted 1:2000 in PBST‐FBS. Cells were then washed twice in PBS, counterstained with Hoechst (Sigma‐Aldrich) diluted 1:1000 in PBS for 10 min at room temperature and washed twice with PBS before analysis.

Pictures of cells were then taken using an Operetta High‐Content Analysis System (PerkinElmer), and single cells were analyzed with the Columbus software using a macro measuring cell body (nucleus with Hoechst) and cytoplasm (with α‐tubulin) (see Table S3 for parameters used to configure the algorithm). The software calculated the distance of elongation from the cell body to their extremity (neurite length) and the number of segments from the cell body. These quantifications were used to determine NED structures.

### 4.7 Calcium imaging

Wide field calcium imaging was performed as previously described (Al‐Mawla et al., 2020; Bosson et al., 2020). For cytosolic Ca^2+^ measurements, MCF‐7 cells (placed 7 days in conditioned medium renewed every 3 days) were loaded with Fura‐2 AM (Molecular Probes, Kd = 0.14 μM) at a final concentration of 2 μM for 30 min at room temperature in Ca^2+^‐containing HBSS (Gibco). All calcium measurements were performed at room temperature in Ca^2+^‐free HBSS (Gibco). Coverslips were mounted on a magnetic chamber (Chamlide). The chamber was placed on a DMI6000 inverted wide‐field microscope (Leica microsystem). Images were acquired with an Orca‐Flash 4.0 Scientific CMOS camera (Hamamatsu) using a 40X oil‐immersion objective. Using a Lambda DG‐4+ filter (Sutter instruments), Fura‐2 AM was excited at 340 and 380 nm and the fluorescent signal emitted measured at 510 nm. Images (1024 × 1024 pixels) were taken with a 5 s interval. Fluorescence ratios (F340 nm/F380 nm) were analyzed with MetaFluor 6.3 (Universal Imaging) after removing background fluorescence. To measure intracellular Ca^2+^ content, ionomycin (Abcam, 120,370) at 1 μM final was added into the medium during acquisition.

### 4.8 METABRIC and GSEA analyses

Data from the Molecular Taxonomy of Breast Cancer International Consortium (METABRIC) (METABRIC Group et al., 2012; Pereira et al., 2016), regrouping gene expression and clinical data, were extracted from the cBioportal website. Expression of four neuroendocrine markers (NEM) in breast tumors from 1904 patients was ranked on a log scale allowing us to discriminate tumors with high or low expression of NEM (expression higher or lower than mean + 1log considered as positive or negative for NEM). With Venn diagrams, patients positive for the four NEMs were considered to be patients with neuroendocrine breast carcinomas (NBC) and the ones negative for the 4 NEMs, non‐NBC. Because all NBC patients were positive for estrogen receptor (ER+), we re‐analyzed the two groups to include only ER+ patients. Thus, further analyses of p53 status, tumor grade, patient age, expression levels, and Gene Set Enrichment Analysis (GSEA) were conducted only on ER+ NBC and ER+ non‐NBC patients. Pre‐Ranked GSEA was performed on ranked lists of gene expression ratio, between average expression in ER+ NBC tumors versus non‐NBC tumors using the GSEA v2.0.13 software with default parameters. All gene set files for this analysis were obtained from the GSEA website (www.broadinstitute.org/gsea/).

### 4.9 Immunoblot

For immunoblot, MRC‐5 were lysed in Laemmli 6X buffer (SDS 2% (m/v), Glycerol 10% (v/v), Tris 125 mM pH, 6.8) with 15% of β‐mercaptoethanol. After determining protein concentration, 20 μg of proteins were heated at 95°C for 10 min, loaded onto 12% gels and separated by SDS‐PAGE in Tris–HCl‐Glycine‐SDS TGS, pH 8.5 buffer (Euromedex). Proteins were then transferred to nitrocellulose membranes (Bio‐Rad) in Tris‐Glycine, pH 8.5 buffer (Euromedex)/ethanol 20% at 4°C for 2 h at a 100 V constant voltage. Membranes were blocked with TBS Tween‐20 0.05% / Milk 5% for 30 min and incubated overnight at 4°C with primary antibodies against CALB1 (Santacruz, sc28285; 1:500 dilution) or against α‐tubulin (Sigma Aldrich, T6199; 1:2000 dilution). Membranes were then washed and incubated with secondary antibody for 1 h at room temperature: anti‐rabbit secondary antibody (Interchim, 711–035‐152) for CALB1 and anti‐mouse secondary antibody (Interchim, 715–035‐150) for α‐tubulin, both diluted at 1:5000. Detection was performed using the ECL kit (Amersham).

### 4.10 ELISA

To analyze IL6 protein levels in cells following 4‐OHT‐induced senescence, human IL6 ELISA kit from Thermo Scientific was used (BMS213‐2). The standard for IL6 was prepared by serial dilutions. Stock solution at 200 ng/ml was diluted to: 6666 pg/ml, 2222 pg/ml, 740.7 pg/ml, 246.9 pg/ml, 82.3 pg/ml, and 27.4 pg/ml, the last dilution point contained only Assay Dilutent. After, 100 μl of each standard and sample were dispatched into a standard 96‐well ELISA plate. The plate was covered and incubated for 2.5 h at room temperature (RT) with gentle shaking. After incubation, the solution was discarded, wells were washed 4 times with 1× washing buffer, and 1× biotinylated antibody was added. The incubation with antibody was conducted for 1 h at RT with gentle shaking. The solution was then discarded, the 4 washes were repeated and streptavidin‐HRP solution added. This step was followed by a 45 min incubation at RT with gentle shaking. After incubation, the solution was discarded and wells were washed for 4 times. TMB substrate was then added and incubated for 30 min at RT in the dark with gentle shaking. Finally, stop solution was added and absorbance was measured at 450 and 550 nm.

### 4.11 Statistical analyses

Means are represented with ± SEM (biological replicates) as indicated in the figure legend. Statistical analyses were performed as indicated in the figure legend using GraphPad Prism 8 software. D'Agostini and Pearson normality test was used before proceeding to any analyses to evaluate the normality of the samples. *p*‐value is indicated on each figure (<0.05: *; <0.01: **; <0.001: ***; <0.0001: ****).

## AUTHOR CONTRIBUTIONS

C.R., X.M., A.H., N.T., A.M, K.Z, F.M.F., F.M, D.G., J.J.M., D.V., H.H.V, and S.D. performed experiments. C.R., X.M., A.H., S.D., N.M., and D.B. designed the experiments and the results were analyzed by all the co‐authors. C.R., X.M., N.M., and D.B. designed the overall study. D.B. and N.M. supervised the work. C.R., N.M., and D.B. wrote the manuscript with input from all authors.

## CONFLICT OF INTEREST

The authors have declared that no conflict of interest exists.

## Supporting information


Figures S1–S8

Tables S1–S3

## Data Availability

The data that support the findings of this study are available from the corresponding author upon reasonable request.
