# Supplementary material for: NF‐κB‐dependent secretome of senescent cells can trigger neuroendocrine transdifferentiation of breast cancer cells
Source: Aging Cell. 2022 Jun 2;21(7):e13632. doi: 10.1111/acel.13632 (PMC9282844; doi:10.1111/acel.13632)

SUPPLEMENTAL FIGURE LEGENDS

FIGURE S1 RAF activation induces senescence and SASP secretion. MRC-5 fibroblasts were infected with a RAF:ER encoding retroviral vector and puromycin selected. The day after seeding, cells were treated with 4-OHT at 100 nM. Six days later, various features of senescent cells were quantified. (a) Cells were fixed and SA-β-Gal assay performed. Percentage of positive cells was calculated (n = 4, unpaired non-parametric Mann-Whitney t-test). (b-d) Relative mRNA levels of (b) p21 CKI, (c) Ki67 proliferation marker and (d) the indicated SASP components (n = 4, unpaired non-parametric Mann-Whitney t-test, mean +/- SEM). (e) IL6 protein levels in the control and SASP supernatants were measured by ELISA (n = 2, mean +/- SD).

FIGURE S2 SASP induces proliferation arrest, but not NED in ER- or p53-mutated breast cancer cells. Six days after treatment with control (CTL) or SASP conditioned media of MRC-5/RAF:ER, (a) T47D and (b) MDA-MB231 were fixed and stained for crystal violet to reveal cell density (left panel) and cell morphology was observed (right panel). (c-g) MCF-7 were transfected with control or p53 directed siRNA and next treated by control or SASP conditioned media. (c) RNAs were extracted and RT-qPCR were performed to quantify p53 mRNA levels (n = 4, one sample t-test, mean +/- SEM). (d) Cells were fixed and stained with crystal violet to visualize cell density. (e) Cells were fixed and SA-β-Gal assay performed. Percentage of positive cells was calculated (n=3, unpaired non-parametric Mann-Whitney t-test, mean +/-SEM). (f) Bright-field micrographs of MCF-7 cell morphology. (g) Cells were fixed, nucleus stained and immunofluorescence against α-tubulin performed before measurements of the indicated

parameters (n = 1200 cells, 3 independent experiments, unpaired non-parametric Kruskal-Wallis test, mean +/- SEM).

FIGURE S3 The ability of SASP to promote NED is conserved with SASP from senescent cells undergoing a genotoxic stress and in prostate cancer cells. (a-d) MCF-7 were treated for six days with conditioned medium from senescence-induced WI-38 using etoposide (etoposide SASP) or control (CTL). (a) Images were acquired to assess MCF-7 cell morphology. (b,c) Immunostaining against α-tubulin was performed. A high content imaging Operetta system was used to automatically acquire images and Columbus software was used to perform analysis of cell shape. (b) Total neurite length per cell and (c) number of segments per cell were quantified (n = 585 cells, 3 independent experiments, unpaired non-parametric Mann-Whitney t-test). (d) RT-qPCR on NEMs SCG2 and CHGB were performed (n = 4, unpaired non-parametric Mann-Whitney t-test, mean +/-SEM). (e-h) LNCaP prostate cancer cells were treated for six days with conditioned media from MRC-5/RAF:ER (SASP) or control (CTL). (e) Images were acquired to assess LNCaP cell morphology. (f, g) Immunostaining against α-tubulin was performed. High content imaging Operetta system was used to automatically take images and Columbus software was used to perform analysis of cell shape. (f) Total neurite length per cell and (g) number of segments per cell were quantified (n = 1401 cells, 3 independent experiments, unpaired non-parametric Mann-Whitney t-test). (h) RT-qPCR on NEM SCG2 and CHGB were performed (n = 6, unpaired non-parametric Mann-Whitney t-test, mean +/- SEM). (i) Six days after treatment with control or SASP conditioned media of MRC-5/RAF:ER, indicated cells were fixed and stained for crystal violet to reveal cell density (upper panel) and cell morphology was observed (lower panel).

FIGURE S4 RELA knockdown abrogates pro-inflammatory SASP, but not the induction of SA-β-galactosidase activity, cell cycle arrest and NF-κB-independent SASP molecules in response to RAF activation. MRC-5/RAF:ER were transfected with a control non-targeting siRNA pool (sictl) or a siRNA pool targeting RELA, one of the NF-kB subunits (siRELA). Cells were treated with 4-OHT after siRNA reverse transfection to activate RAF and induce senescence. Six days after treatment, (a) RELA knockdown was validated by RT-qPCR on RELA mRNA expression. (b) Expression of NF-κB-dependent SASP interleukins IL6 and IL8 was also assessed, as well as (c) the percentage of cells positive for SA-β-galactosidase activity and (d) mRNA levels of p21, NF-κB-independent molecule (e) BMP-2 and (f) SPP1 by RT-qPCR (n = 4, unpaired non-parametric Mann-Whitney t-test, mean +/-SEM).

FIGURE S5 IκBα super-repressor inhibits pro-inflammatory SASP and impairs the NED induced by this SASP. MRC-5/RAF:ER stably expressing IκBα super-repressor (SR) were treated with 4-OHT to activate RAF and induce senescence. Six days after treatment, (a) IκBα SR expression was validated by RT-qPCR on IκBα mRNA expression. (b) Expression of NF-κB-dependent SASP interleukins IL6 and IL8 were assessed. (c) p21 mRNA levels, as senescence marker, and (d) BMP-2 and SPP1, as NF-κB-independent SASP were assessed by RT-qPCR (n = 3, mean +/-SEM). (e-f) MCF-7 were treated by the control or SASP conditioned media. (e) Bright-field micrographs of MCF-7 cell morphology. (f) After staining, total neurite length per cell and the number of segments per cell were quantified (n = 3, unpaired non-parametric Kruskal-Wallis test, mean +/- SEM).

FIGURE S6 Effect of various SASP on Ca^{2+} levels. (a) Resting cytosolic Ca^{2+} levels in MCF-7 were measured with the ratiometric probe Fura2-AM after 3 or 6 days (n = 194 for CTL and n = 205 for SASP at D3 and n = 181 for CTL and n = 91 for SASP at D6, 3 independent experiments, non-parametric Mann-Whitney t-test, mean +/- 95% of confidence) of CTL or SASP treatment. (b) SASP, or its CTL, were prepared from MRC-5/RAF:ER stably expressing or not the IκBα super-repressor (SR). Total intracellular Ca^{2+} contents in MCF-7 cells were measured with Fura2-AM probe after 3 days of CTL or SASP treatment (n = 218 for all conditions, 2 independent experiments, non-parametric Kruskal-Wallis test, mean +/- 95% of confidence). (c) CTL or SASP were applied for 3 days to MCF-7 cells previously transfected with control (sictl) or p53 (sip53) targeted siRNA and next total intracellular Ca^{2+} contents were measured using Fura2-AM (n = 145 for all conditions, 2 independent experiments, non-parametric Kruskal-Wallis test, mean +/- 95% of confidence).

FIGURE S7 Analyses of gene expression from breast cancer patients reveal that few patients present high expression levels of NEM. METABRIC data of breast tumor patients (gene expression for 1,904 tumors) were extracted and analyzed. Expression levels of four neuroendocrine markers (SCG2, CHGB, CHGA and synaptophysin, SYP) in each breast tumor were ranked from lower to higher expression per patient. Cut-off representing the mean log value of the total expression level for each NEM + 1 was chosen. Patients having lower NEM expression levels than the cut-off were considered to be negative for the NEM, or positive if they displayed a higher expression level than the cut-off.

FIGURE S8 Recombinant IL6 treatment induces neurite-like morphology in LNCaP prostate cancer cells, but not in MCF-7 breast cancer cells. (a) LNCaP cells were treated with recombinant IL6 at 20 ng/mL. After 7 days, images were acquired to assess LNCaP cell morphology. (b) MCF-7 cells were treated with recombinant IL6 at 20 ng/mL. After 7 days, images were acquired to assess MCF-7 cell morphology.

SUPPLEMENTARY 1

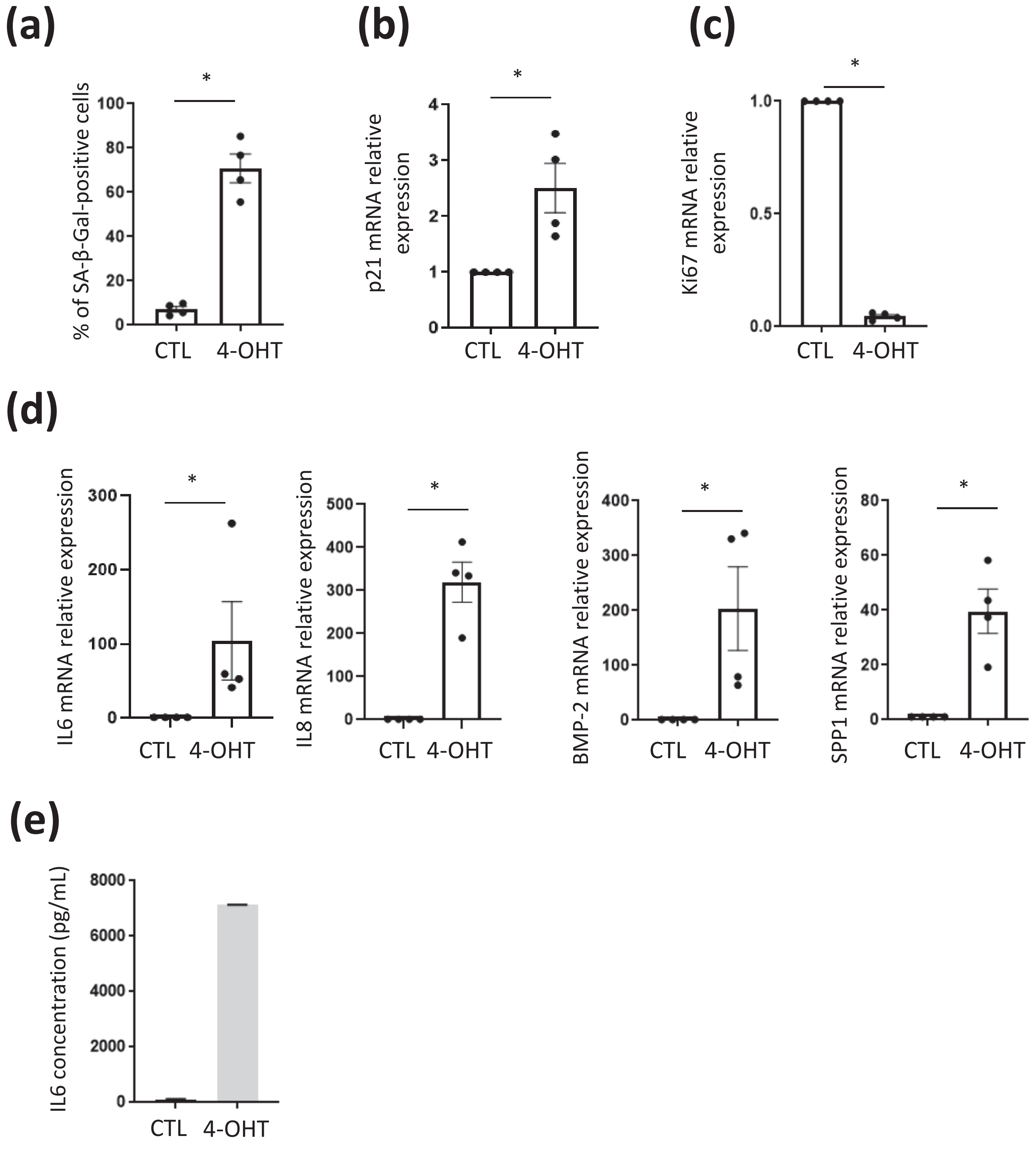

SUPPLEMENTARY 2

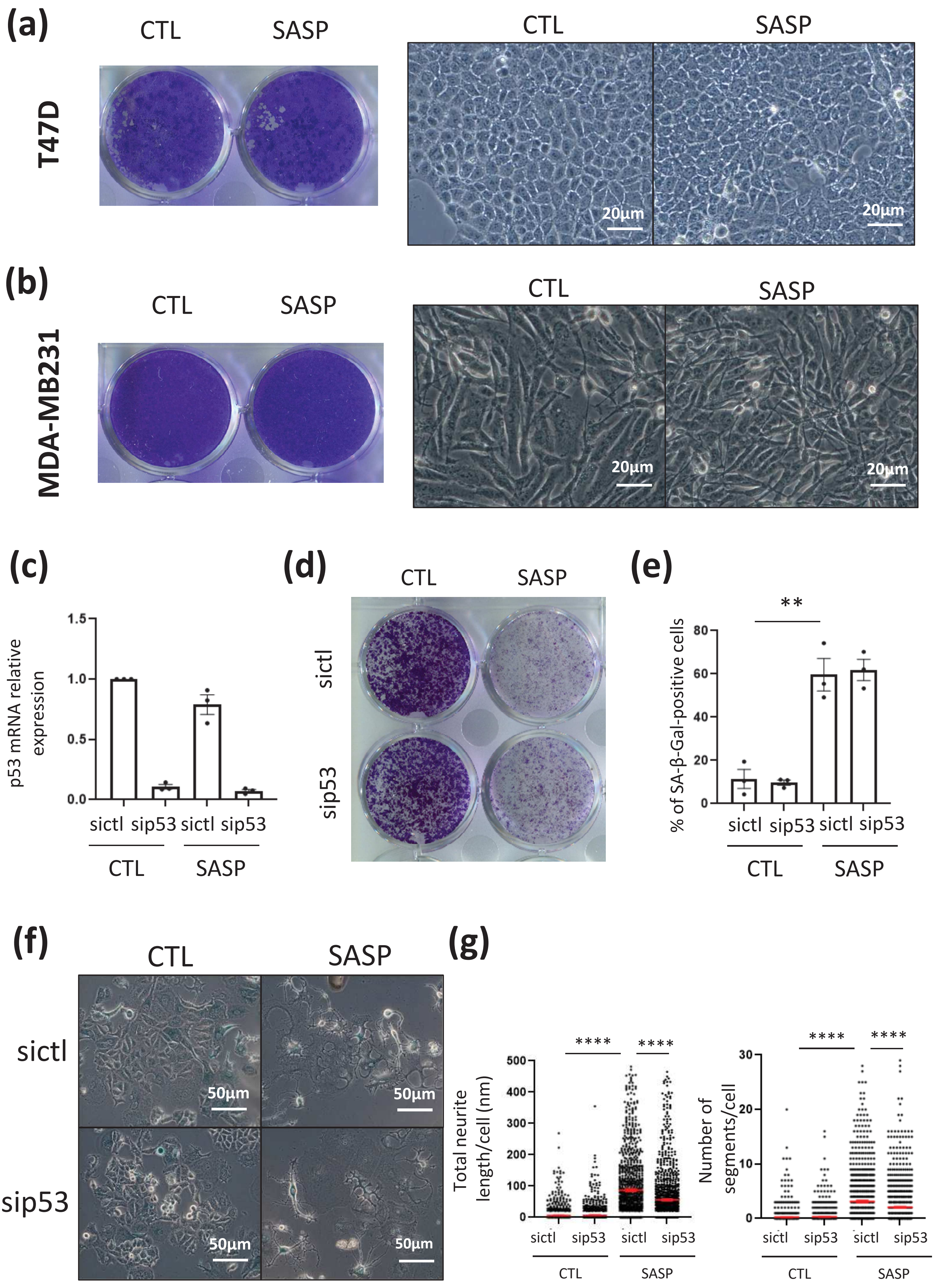

SUPPLEMENTARY 3

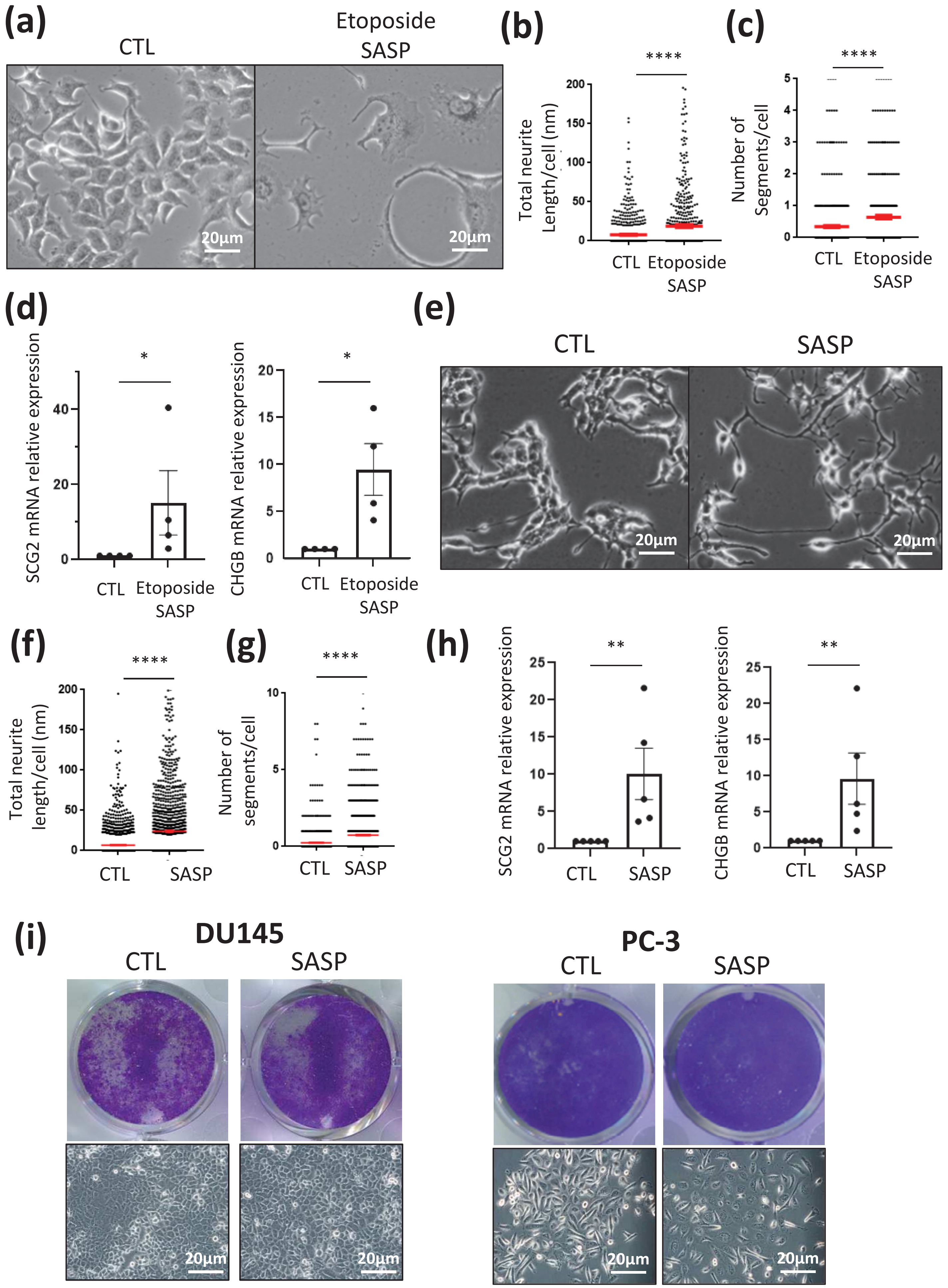

SUPPLEMENTARY 4

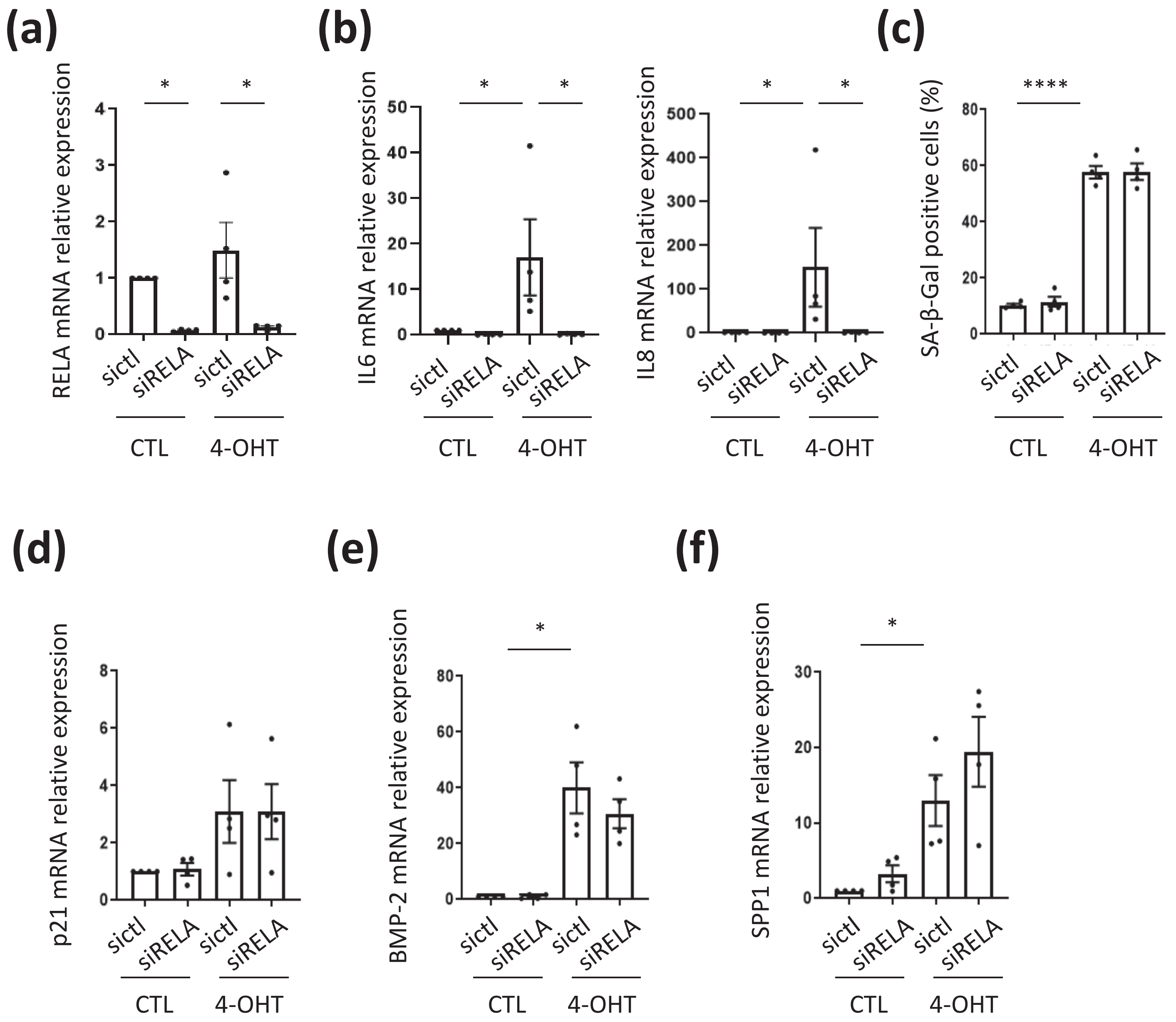

SUPPLEMENTARY 5

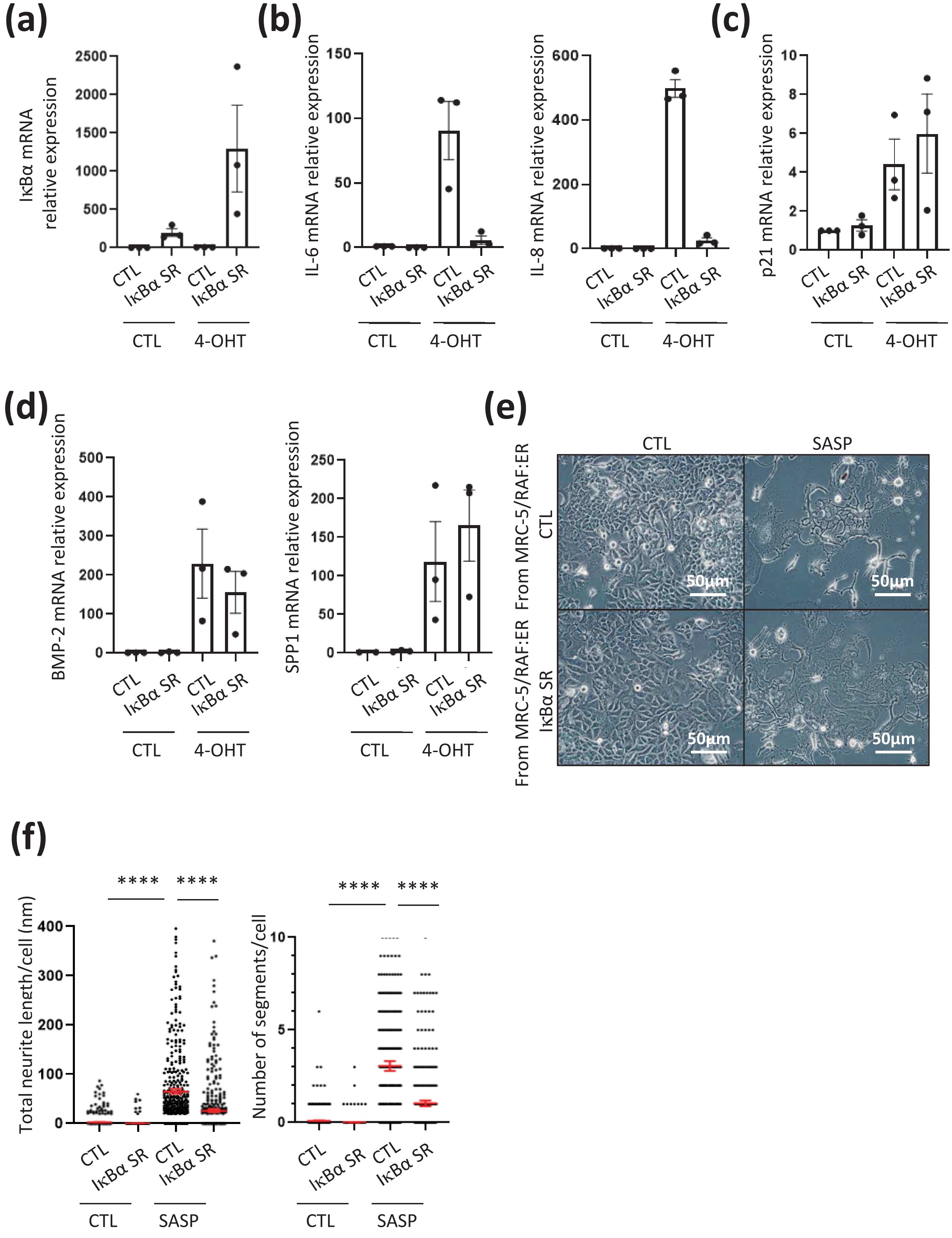

SUPPLEMENTARY 6

(a)

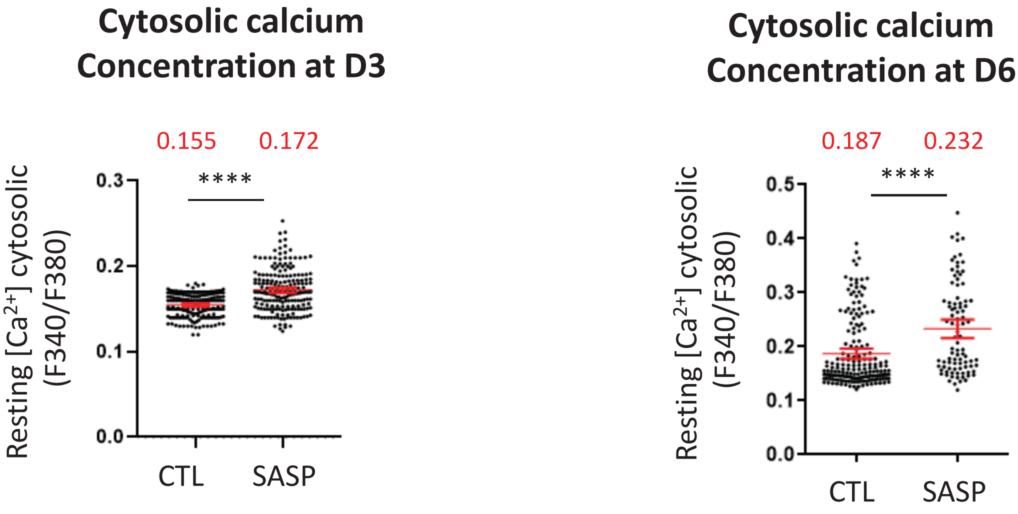

(b)

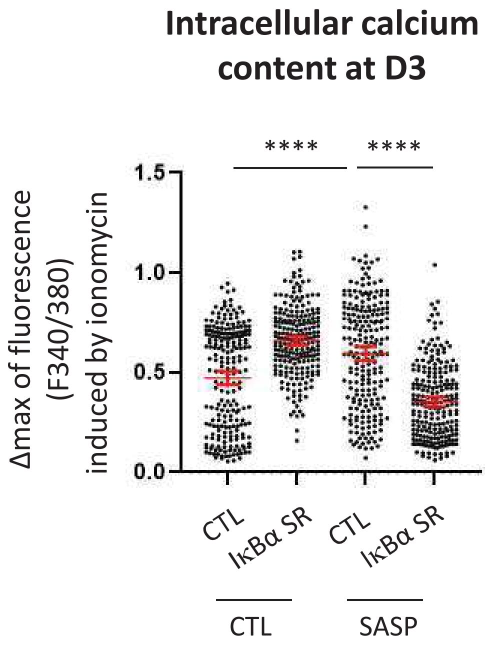

(c)

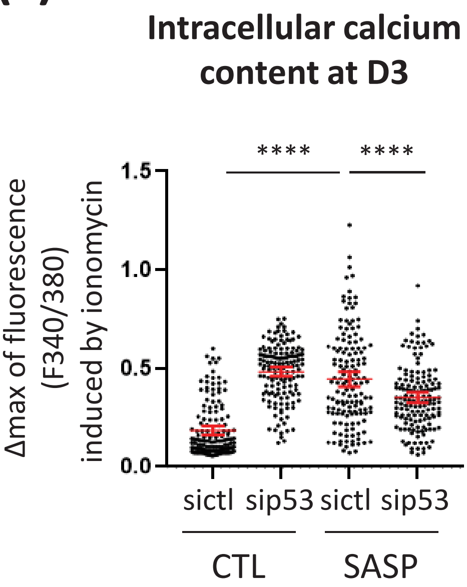

SUPPLEMENTARY 7

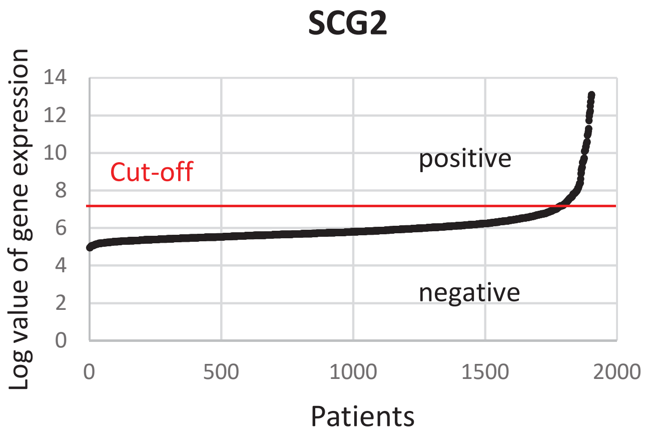

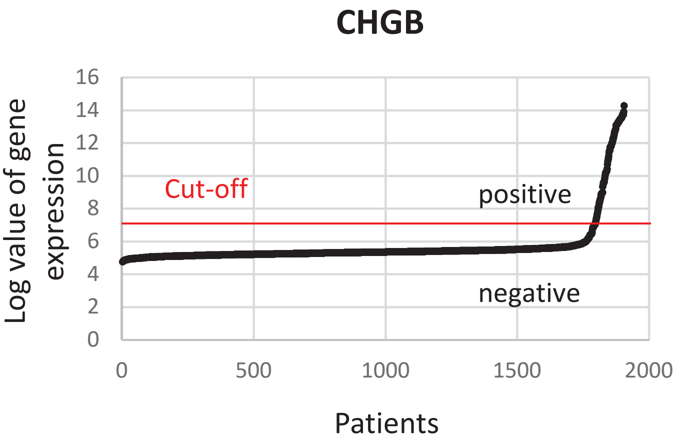

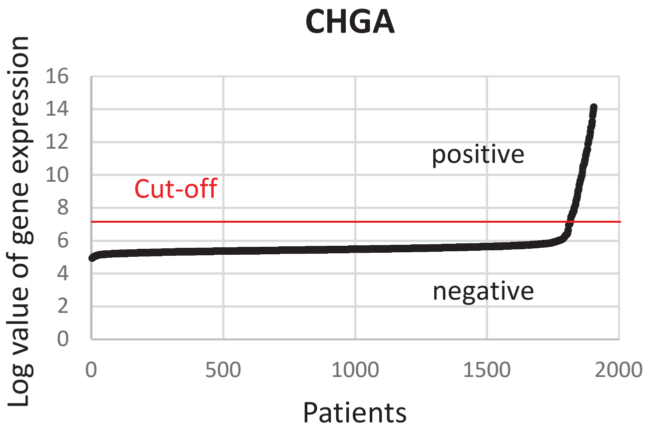

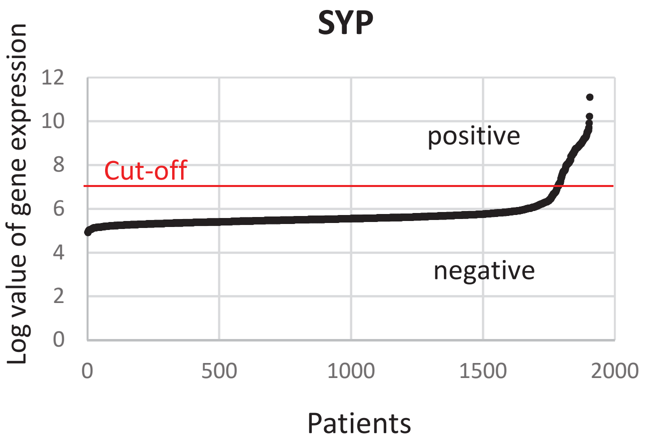

SUPPLEMENTARY 8

(a)

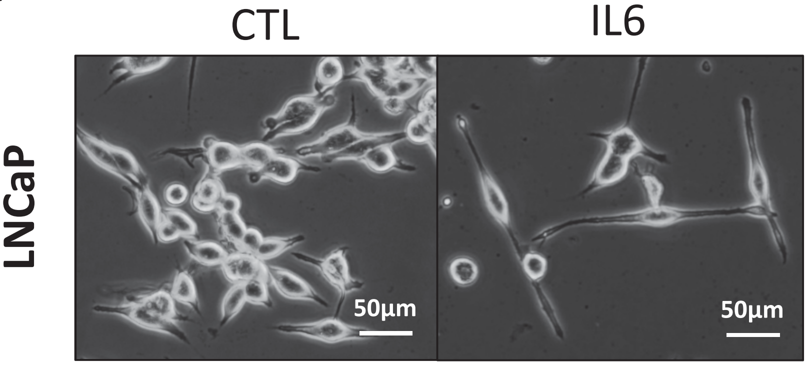

(b)

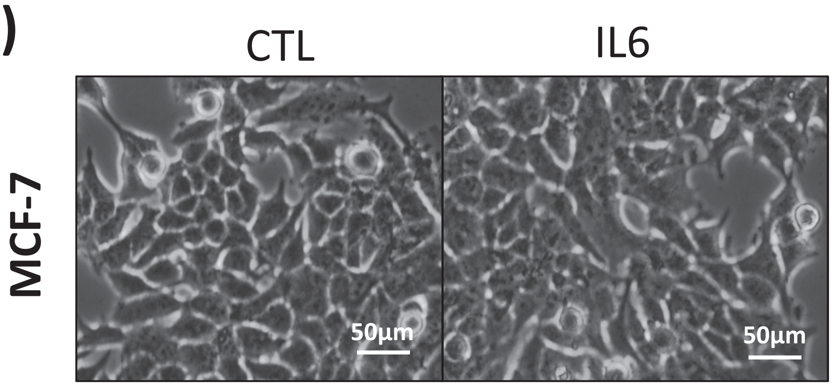

Supplement: Supplementary file 1 — Figures S1–S8 [file ACEL-21-e13632-s001.pdf]
