# Supplementary material for: NF‐κB‐dependent secretome of senescent cells can trigger neuroendocrine transdifferentiation of breast cancer cells
Source: Aging Cell. 2022 Jun 2;21(7):e13632. doi: 10.1111/acel.13632 (PMC9282844; doi:10.1111/acel.13632)
Supplement: Supplementary file 2 — Tables S1–S3 [file ACEL-21-e13632-s002.pdf]

Supp. Table 1. siRNA sequences used for reverse transfection.

Name	Accession number NM_	Sequences
sictl		UGGUUUACAUGUCGACUAA
sictl		UGGUUUACAUGUUGUGUGA
sictl		UGGUUUACAUGUUUUCUGA
sictl		UGGUUUACAUGUUUUCCUA
siRELA	46430498	GGAUUGAGGAGAAACGUAA
siRELA	46430498	CCCACGAGCUUGUAGGAAA
siRELA	46430498	GGCUAUAACUCGCCUAGUG
siRELA	46430498	CCACACAACUGAGCCCAUG
siP53	000546	GAAAUUUGCGUGUGGAGUA
sip53	000546	GUGCAGCUGUGGGUUGAUU
sip53	000546	GCAGUCAGAUCCUAGCGUC
sip53	000546	GGAGAAUAUUUCACCCUUC

Supp. Table 2. Primers sequences and UPL used for qPCR.

Name	Forward	Reverse	Probe
GAPDH	AGCCACATCGCTCAGACAC	GCCCAATACGACCAAATCC	UPL #60
IL8	AGACAGCAGAGCACACAAGC	ATGGTTCCTTCCGGTGGT	UPL #72
P21	TCACTGTCTTGTACCCTTGTGC	GGCGTTTGGAGTGGTAGAAAT	UPL #32
IL6	GATGAGTACAAAAGTCCTGATCCA	CTGCAGCCACTGGTTCTGT	UPL #2
SPP1	GAGGGCTTGGTTGTCAGC	CAATTCTCATGGTAGTGAGTTTTCC	UPL #18
BMP-2	CGGACTGCGGTCTCCTAA	GGAAGCAGCAACGCTAGAAG	UPL #49
CALB1	AAGATCCGTTCGGTACAGCTT	CTGAAGGATCTGTGCGAGAA	UPL #58
SCG2	GGGAGGAATATGCTGTGGAG	AGCCATGTTTGAAAGATTTCCT	UPL #53
CHGB	CCCCTGAGGACCTGGAGT	GGTCTTGGAGCCCTGTATTCT	UPL #79
TP53	AGGCTTGGAACTCAAGGAT	CCCTTTTTGGACTTCAGGTG	UPL #12
KI67	TCAAGGAACTGATTCAGGAGAAG	GTGCACTGAAGAACACATTTCC	UPL #32
RELA	CTGGCTTGGGGACAGAAG	TCATGAAGAAGAGTCCTTTCAGC	UPL #39
IKBA	GCCGGACTGCCCTTCACCTC	GTATCCGGGTGCTTGGGCGG	No (SYBR)

Supp. Table 3. Parameters used for quantification of neurite-like structures on Columbus software

	Input Population	Method
**Channel : Hoechst « Nuclei »**	Find nuclei	**Method : B**<br>Common Threshold : 0.4<br>Area : > 30 µm²<br>Splitting Coefficient : 6<br>Individual Threshold : 0.3<br>Contrast : > 0.1
	Calculate Morphology Properties	**Method : Standard Area**<br>Ratio Width to Length<br>**Method : Filter by Property**<br>Nucleus Ratio Width to Length : > 0.5
**Channel : Alexa 555 « Cell »**	Find cytoplasm	**Method : F**<br>Membrane Channel : Alexa 555<br>Individual Threshold : 0.15
	Calculate Morphology Properties	**Method : Standard Area**
**Channel : Hoechst « Nuclei »**	Select Population	**Method :** Filter by Property<br>Cell Area [µm²] : < 8000<br>**Method : Common Filters**<br>Remove Border Objects<br>Region : Cell<br>**Method : Resize Region [µm/px]**<br>Outer Border : -15 px<br>Inner Border : INF px
**Channel : Alexa 555 « Cell »**	Find neurites	**Method : CSIRO Neurite Analysis 2**<br>Smoothing Width : 3 px<br>Linear Window : 11 px<br>Contrast : > 7<br>Diameter : ≥ 7 px<br>Gap Closure Distance : ≤ 9 px<br>Gap Closure Quality : 0<br>Debarb Length : ≤ 15 px<br>Body Thickening : 5 px<br>Tree Length : ≤ 20 px
